# Intra-abdominal Testicular Torsion: A Case Report

**DOI:** 10.7759/cureus.112905

**Published:** 2026-07-18

**Authors:** Oualid Bounouar, Anouar El Moudane, Mohammed Ramdani, Ahmed Jdaini, Ali Barki

**Affiliations:** 1 Urology, Mohammed VI University Hospital Oujda, Oujda, MAR

**Keywords:** abdominal pain, case report, ectopic testis, testicular torsion, undescended testis

## Abstract

The torsion of an ectopic testis is a rare urological condition encountered only infrequently in clinical practice, and its symptoms may be misleading and interpreted as signs of other common conditions. We report an unusual case of a 20-year-old male patient who sought emergency medical care for acute abdominal pain. On examination, an empty left hemiscrotum was found, and an immediate (CT) scan of the abdomen showed torsion of an intra-abdominal testis. The patient underwent urgent surgical exploration, during which the ectopic testis was found to be viable and was successfully preserved and fixed in a suprascrotal position by orchiopexy. The salvage rates for torsion of the ectopic testis are low but a clinical suspicion and early diagnosis may salvage the testis. It is essential that any young man presenting to the emergency department with abdominal pain undergo a full genitourinary examination, including testicular examination.

## Introduction

Spermatic cord torsion constitutes a urological emergency that requires immediate diagnosis and surgical intervention. Testicular torsion is an acute vascular emergency caused by twisting of the spermatic cord, leading to compromised testicular perfusion and, if left untreated, irreversible ischemic damage. The severity of ischemia is primarily determined by the duration of torsion and the degree of spermatic cord rotation [1]. However, despite being described in the urological literature, torsion of an ectopic testis remains an infrequently recognized clinical condition. Its atypical presentation can mimic numerous causes of acute abdominal pain, posing a diagnostic challenge. Because early recognition is difficult, diagnostic delays occur frequently and markedly diminish the likelihood of testicular salvage.

Ectopic testis is a rare congenital anomaly in which the testis exits the external inguinal ring but deviates from the normal pathway of descent and settles in an abnormal extratesticular location, such as the superficial inguinal pouch, perineum, femoral region, suprapubic area, or inner thigh. This condition differs from cryptorchidism, in which the testis remains along the normal descent route [2]. We present a case of torsion of an undescended intra‑abdominal testis in a 20‑year‑old man to highlight the diagnostic challenges and the importance of early recognition of this urologic emergency.

## Case presentation

**Figure 1:**
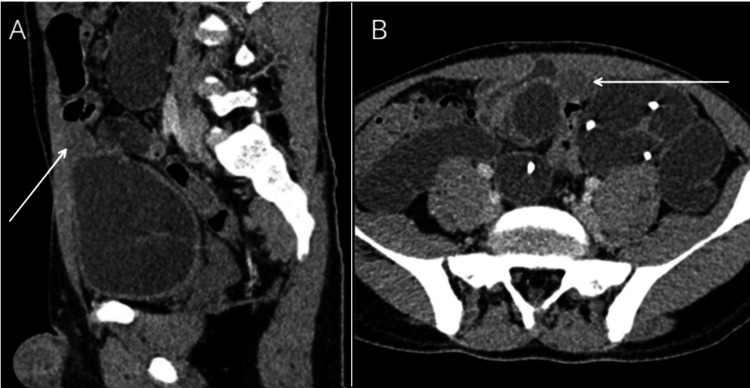
CT scan demonstrates a supravesical mass consistent with an ectopic testis. (A) Sagittal CT scan showing the ectopic testis located above and anterior to the urinary bladder, delineated as an oval soft-tissue density clearly separated from midline pelvic structures. (B) Axial CT image demonstrating the ectopic testis as a well-defined, oval soft-tissue mass situated supravesically. CT: computed tomography.

A 20-year-old man presented to the emergency department of our hospital, with two hours of severe abdominal pain, which was sudden without any precipitating factor and associated with episodes of vomiting. He had a history of left undescended testis (UDT). Initial vital signs were as follows: temperature, 37°C; heart rate, 85; blood pressure, 119/78. Physical examination revealed the absence of left testis in the left hemiscrotum, and no inguinal masses were seen or palpated. His abdominal exam revealed a soft, non-tender, non-distended abdomen without masses, guarding, or rebound. The right hemiscrotum was normal with a normally placed testis. The rest of the general physical examination was unremarkable, and laboratory tests were unremarkable. An immediate abdominopelvic computed tomography (CT) scan was ordered and demonstrated an intra-abdominal supravesical mass, most likely an ectopic testis (Figure 1). Differential diagnoses, including appendicitis and intestinal obstruction, were excluded.

**Figure 2:**
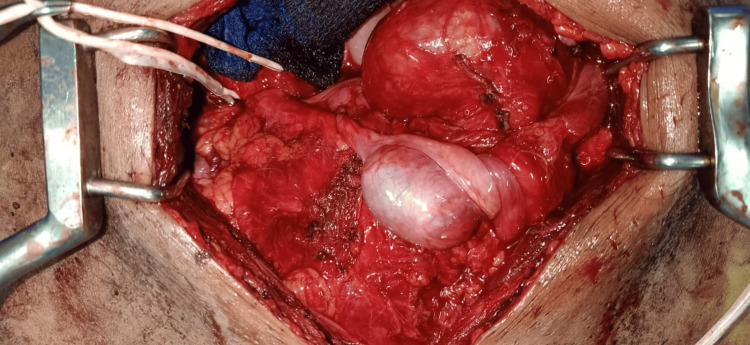
Intraoperative photograph of the anterior abdominal wall following a midline incision, demonstrating ectopic testicular torsion.

The patient was taken for exploration in the dorsal position on the operating table under general anesthesia. The decision was made to make a midline incision to allow adequate exploration of the abdominal cavity in case another diagnosis was present. During surgery, torsion of the left testis was found with two spiral turns just behind the abdominal wall. Fortunately, the diagnosis was made early, and the testis was viable (Figure 2).

**Figure 3:**
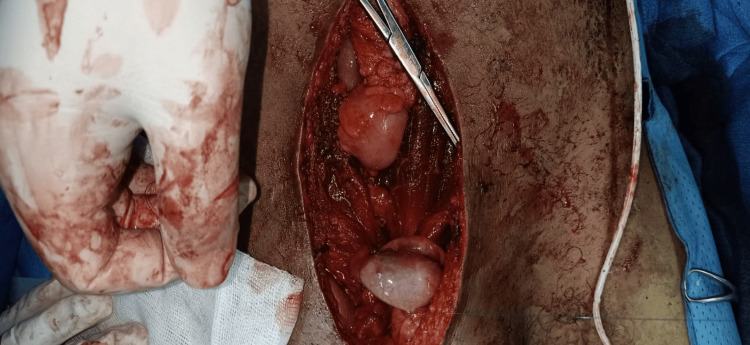
Intraoperative view following fixation of the left testis.

Detorsion and fixation of the left testis was performed. The spermatic cordon was not long enough to be brought down to the scrotum, so the testis was fixed in a suprascrotal position, where it was just palpable (Figure 3). The postoperative course was uneventful, and the patient was discharged on the third postoperative day with analgesic treatment and antibiotic. The patient was seen 10 days after surgery for a checkup.

Fortunately, the CT scan was performed early, which allowed the patient to be taken to the operating room quickly, and the testis was preserved.

## Discussion

Cryptorchidism is the most common genitourinary abnormality in male neonates, with a reported incidence of 1%-4.6% in term infants and up to 45% in preterm infants [3,4]. The UDT may be located anywhere along its normal pathway of descent or in an ectopic position. Cryptorchidism is defined as the failure of testicular migration along the normal descent route, most commonly within the inguinal canal (approximately 70%), while the ectopic testis is defined by the presence of the testis out of its usual path [5]. The most serious complications of UDT or ectopic testis are a high rate of infertility, an elevated risk of testicular malignancy, and testicular torsion [6]. Orchidopexy should be done between 6 and 18 months. Early surgical correction is recommended to preserve fertility potential and reduce the long-term risk of testicular malignancy [7].

The pathophysiology of spermatic cord torsion in UDT is not fully understood, but several mechanisms have been suggested. These include abnormal cremasteric muscle activity and a relative increase in testicular size compared with its mesenteric attachment, a factor that may also account for the observed association with testicular tumors [8].

Ectopic testicular torsion is a rare clinical entity frequently associated with a misleading clinical presentation, which may result in diagnostic delay and ischemic necrosis of the involved testis. The clinical symptoms of undescended testicular torsion are abdominal pain, malaise, and nausea or vomiting. Physical exam findings depend on the location of the UDT. Approximately 76% of UDT are palpable [9], with an empty ipsilateral hemiscrotum [8]. Therefore, any abdominal pain in a patient with a history of ectopic testis should be considered as a testicular torsion. The prognosis for testicular preservation in cryptorchid torsion remains poor, with reported salvage rates of approximately 10%, all depending on the delay of intervention [10]. Early clinical recognition and timely diagnostic assessment are critical determinants of successful salvage of the ectopic testis. The peak incidence of torsion has been reported around puberty and in the first year of life [11], but no age is exempted, as was seen in our patient who was 20 years old.

Doppler ultrasonography, computed tomography, and technetium Tc-99m scrotal scintigraphy can provide valuable diagnostic information; however, these investigations should not delay prompt surgical exploration when testicular torsion is suspected [12].

Whether prophylactic contralateral orchidopexy should be routinely performed during the surgical management of ectopic testicular torsion remains controversial, given the limited available evidence. However, the majority of authors recommend orchidopexy of the contralateral testis following orchiectomy to prevent the loss of the solitary functioning testis [13]. In our case, contralateral orchidopexy was not performed after detailed counseling regarding the potential risk of future torsion; the patient declined the procedure despite a discussion of the possible benefits of prophylactic fixation reported in the literature.

## Conclusions

Ectopic testicular torsion is an uncommon clinical entity that should be considered by clinicians in the differential diagnosis of abdominal pain. This underscores the importance role of prompt clinical evaluation, including careful abdominal and scrotal examination, in raising suspicion for this uncommon condition. Although imaging studies may provide valuable diagnostic support, they should not delay urgent surgical exploration when torsion is suspected, as delayed management may significantly compromise testicular viability. Early diagnosis and rapid management are essential to preserve the testis.
